# The importance of eliciting stakeholders’ system boundary perceptions for problem structuring and decision-making

**DOI:** 10.1016/j.ejor.2021.12.029

**Published:** 2022-10

**Authors:** Irene Pluchinotta, Giuseppe Salvia, Nici Zimmermann

**Affiliations:** Institute for Environmental Design and Engineering, The Bartlett Faculty of The Built Environment, University College London, UK

**Keywords:** Problem structuring methods, System dynamics, System perception, Causal Loop Diagrams, Boundary Critique

## Abstract

Differences in system boundaries and problem framings are unavoidable in multi-organisational decision-making. Unstructured problems, such as the grand challenges, are characterised by the existence of multiple actors with different perspectives and conflicting interests, and they require a coordinated effort from multiple organisations. Within this context, this paper aims to understand stakeholders’ perceptions of system boundaries and problem framings, and their potential effects on decision-making by systematically comparing different stakeholder groups’ causal maps around the same shared concern. Bridging notions from Operational Research, System Dynamics and Organisational Studies, the comparison is based on a novel type of thematic analysis of Causal Loop Diagrams (CLDs) built with each stakeholder group on their perceptions of a given system. The proposed integrated approach combines qualitative with quantitative analysis, such as the centrality of the variables and the structure of the CLDs. Such CLDs comparison provides an intuitive way to visualise differences and similarities of the thematic clusters of variables, underlining factors influencing the shared concern. This could be considered a starting point for more shared understanding as well as more integrated holistic perceptions of the system and, consequently, a more systemic decision-making. Furthermore, for the sake of replicability, this paper also presents a qualitative participatory System Dynamics modelling process aimed to define the key aspects of a problem for each group of stakeholders to support a collaborative multi-organisational decision-making process. The research is based on the activities carried out for an urban regeneration case study in Thamesmead, London, United Kingdom.

## 1 Introduction

Scholars increasingly encourage research tackling grand challenges, namely formulations of global problems that can be plausibly addressed through collaborative effort (Ferraro, Etzion & Gehman, 2015). The most widely adopted grand challenges are those related to the Sustainable Development Goals of the United Nations. By their very nature, sustainability challenges require co-ordinated and sustained effort from diverse stakeholders and organisations to pursue the adoption of less conventional approaches to tackle large problems (George et al., 2016). Unstructured problems, such as environmental ones, are characterised by the existence of multiple actors, various perspectives, important intangibles, and key uncertainties (Rosenhead & Mingers, 2000). It is difficult to talk of “problems” as such, since the very construction of the situation as being a problem of a particular type is a result of the process of problem structuring rather than being a given starting point (Mingers & Rosenhead, 2004). It may therefore be better to talk of different aspects or dimensions of a problem situation, rather than different types of problem (Checkland, 1981). It is thus important to consider the complexity of multi-level and multi-organisational decision-making contexts, in which stakeholders and decision-makers with different, and often conflictual, understanding, objectives and values are required to work together to achieve sustainable targets.

Differences in system boundaries and problem framings are unavoidable in multi-organisational decision-making processes (Churchman, 1970; Midgley, 2000; Ulrich, 1983). These differences can lead to a polarisation of viewpoints, reducing the effectiveness of a strategy/policy/decision (Giordano, Brugnach & Pluchinotta, 2017) or to the misperceptions of the system, creating misperceptions of needs and distort evaluation (Kim, 2008). Indeed, under the presence of ambiguity or discrepancies in the way in which a certain situation is interpreted, it may not be clear if a situation is problematic or not, or if there is a problem, what the problem is, or whose problem it is, or what actions path should be taken to deal with it (e.g., Brugnach & Ingram, 2012; Brugnach et al., 2011; Giordano et al., 2020).

Each stakeholder sees the system from their own point of view and may thus, first, fail to detect unintended consequences that traverse this narrow system boundary, and second, only consider solutions that sit within the perceived boundary. This is because, too often, narrow perspectives drive decisions (e.g., Pluchinotta et al., 2018; Shrubsole et al., 2019). Ill-designed solutions create a multitude of unintended consequences and are thus not sustainable also in the long run and for a multitude of stakeholders (Ferretti, Pluchinotta & Tsoukiàs, 2019). This is especially the case where enhancing the accuracy of decision-makers’ understanding of either a situation or a system is one of the goals of decision support activities (Schaffernicht & Grösser, 2011). Whole-system perspectives that use a broad system boundary are required to enhance the chance of detecting unintended consequences that are distant in space or in time and to improve decision-making (Churchman, 1970).

Decision-makers’ knowledge, or mental model, of the system they manage provides the raw material for debate and discussion (Morecroft, 1988). Expanding this knowledge, broadening boundary perceptions, is pivotal in case of a multi-stakeholder context (Kim, 2008), such as sustainability, particularly for the potential changes it can trigger in decision-makers’ perceptions of where problem boundaries lie (Bérard, Cloutier & Cassivi, 2017). Defining problem frames or system boundaries indicates what issues, solutions and stakeholders are to be included in the decision–making process (Bérard et al., 2017). Individuals necessarily have ‘boundaries’, and the challenge of decision support is not to settle on a specific solution but rather to favour more systemic thinking during the decision-making process (Midgley & Richardson, 2007). Indeed, by assessing individual and group understanding of local complex systems, Valcourt et al. (2020) have discovered that alignment of factors, causal links, and feedback loops identified by participants improves when individuals are convened in a group setting.

A stakeholder group’s problem frame and system boundary are affected by socially structured patterns of attention (Ocasio, 1995; Ocasio 1997). A number of studies in the management area have shown the importance of changing foci of attention if changes in decisions and actions are to result (Cho & Hambrick, 2006; Eggers & Kaplan, 2009; Nadkarni & Barr, 2008; Ocasio, Laamanen & Vaara, 2018). Thus, an analysis of problem frames and system boundaries, including causal interactions and feedback loops, could be a useful input to multi-stakeholder decision-making and change processes.

As underlined by Schaffernicht and Grösser (2011, 2014), research lacks a comprehensive method to compare mental models, which include the essential conceptual elements of such systems. Furthermore, this paper argues that mathematical approaches to compare causal maps are not always suitable when a thematic comparison of different perceptions is needed for supporting multi-stakeholder decision-making.

Within this context, our research aims to understand stakeholders’ perceptions of system boundaries and their potential effects on decision-making by systematically comparing different stakeholders’ system maps around the same issue. The comparison is based on a novel type of thematic analysis of Causal Loop Diagrams (CLDs) constructed with each stakeholder group on their perceptions of the system, combining qualitative with quantitative analysis, such as the centrality of the variables and the loops of the CLDs. The paper also investigates how it is possible to support organisational stakeholders through system modelling to create shared understanding of the system and adopt a systems perspective and solutions that fulfil a multitude of sustainability criteria. Lastly, the paper describes a qualitative participatory System Dynamics (SD) modelling process aimed to define the key aspects of a problem for each group of stakeholders, to support a more inclusive multi-organisational decision-making process.

The research is based on activities carried out within a wider participatory SD modelling process for an urban regeneration case study based in Thamesmead, South-East London, United Kingdom. Specifically, a set of modelling workshops for building CLDs around a jointly-identified shared concern were carried out (see Ostanello & Tsoukiàs, 1993, and Pluchinotta et al., 2019, for the definition of a shared concern). Each CLD represents a stakeholder groups’ perception of the system boundaries under consideration. Using a novel method that combines thematic analysis with model analysis methods, we systematically analysed and then compared all CLDs and presented results to the stakeholders afterwards, to align the differences within a collaborative and multi-stakeholder decision-making process.

Our work is guided by theory and concepts from both SD (Kim, 2008; Morecroft, 1988) and Operational Research (OR), specifically Problem Structuring Methods (Rosenhead & Mingers, 2000) and Boundary Critique (Midgley, Munlo & Brown, 1998). Furthermore, due to its focus on stakeholders’ perceptions, this work is theoretically inspired by concepts of organisational attention and framing (Kaplan, 2008; Ocasio, 2011) from Organisational Studies. The latter is considered important because people are oblivious to issues and solutions outside of their attention (Zimmermann, 2011). This represents an additional effort to bridge the three research communities whose methodological synergies are rarely investigated, despite their potential impact on decision-making.

The remainder of the paper is organised as follows: after an overview of methodologies for comparing causal maps (Section 2), we present the novel methodology for building and comparing CLDs within a collaborative and multi-stakeholder decision-making process (Section 3), and then apply it within the case study (Section 4). Section 5 discusses the main findings and concludes the paper.

## 2 Understanding the differences

In this section we review the literature of (i) mapping mental models via Causal Loop Diagrams (CLDs) vs. Cognitive maps (CMs), (ii) comparing causal maps, (iii) the theoretical views of Boundary Critique and (iv) the Attention-based View to understand stakeholders’ differences in the perceptions of system boundaries.

### 2.1 Causal maps of mental models

A mental model is an abstract representation of a system in the mind of an individual (Forrester, 1961, 1992). It reflects the beliefs, values, and assumptions that the individual personally holds, underling the reasons for decision-making (Maani & Cavana, 2007; Schaffernicht & Grösser, 2011). Mental models are commonly used in both SD and OR research communities and represented in CMs and CLDs.

For the sake of clarity, key definitions from both domains for CMs and CLDs will be recalled. The term “cognitive mapping” is used to describe the task of mapping a person’s or group’s mental model of a problem or situation (Eden, 2004). Each CM thus represents both an individual or a group’s beliefs concerning a particular domain at a point in time (Axelrod, 1976; Langfield-Smith & Wirth, 1992). It is characterised by constructs (usually phrases with variables) as nodes, and causal links (positive or negative) representing events or actions (Schaffernicht, 2007). Eden (2004) underlines that CMs are characterised by a hierarchical structure which is most often in the form of a means/ends graph with goal type statements at the top of the hierarchy. This hierarchical form is often informed by some circularity in which a chain of means and ends loops back (Eden, 2004).

Besides CMs, CLD are well-known examples to visualise individuals or teams’ mental models (e.g., Vennix, 1996; Kim, 2009). CLD is one of the core concepts of SD, and it maps the feedback structure of systems (Sterman, 2000), rather than being concerned with hierarchical form (Eden, 2004). A CLD consists of variables connected by causal links with an assigned polarity, denoting the causal influence amongst variables, and reinforcing and balancing feedback loops (Meadows et al., 1972). CLDs are commonly used to communicate the important feedback responsible for a certain problem within a decision-making process (Lane, Munro & Husemann, 2016). The SD approach rests on the premise that the dynamic behaviour as exhibited by various systems is due to the presence of causal loops of interdependence of various variables in a system (Seth, Seth & Sushil, 1994). The causal structure is not separated from its behaviour, which is one principle of SD (Grösser & Schaffernicht, 2012).

Arguably, also the cognitive mapping approach deals with dynamic situations; it uses diagramming techniques to structure problems and to articulate the causal understanding of problem owners (Ackermann & Eden, 2011). However, CLDs are event-based representations capturing the dynamicity of a certain system thanks to the feedback approach, not only challenging conventional event-orientated thinking but also producing dynamics consistent with the observed problem (Morecroft, 1988).

In conclusion, it has already been discussed how CMs and CLDs are based on similar basic principles of causal mapping (e.g., Howick, Ackermann & Andersen, 2006; Lane, 1999; Rosenhead & Mingers, 2000) and most importantly how both share a focus on people’s perceptions (Rouwette, Vennix & Felling, 2009). For instance, Forrester (1994) underlines that the conceptualisation phase of SD has much in common with soft OR methodologies, specifying that SD is disciplined by an organizing framework that leads to model formulation and simulation.

With the differences and similarities between the two approaches in mind, it is worth pointing out that both CLDs and CMs are directed graphs, namely networks of nodes and arrows where all the edges are directed from one vertex to another (Harary, Norman & Cartwright, 1965), and the direction of the arrow implies believed causality (Eden & Ackermann, 2004). Therefore for the purpose of this paper, in our comparison of causal maps, we rely on insights about both CLDs and CMs.

### 2.2 Comparing causal maps

As causal maps allow to represent visually our understanding of mental models, a comparison of causal maps can shed light on important similarities and differences between different groups’ thinking. The comparison of different causal maps improves the understanding of how people comprehend, interpret, and subsequently influence the system in which they operate (Schaffernicht & Grösser, 2014).

Grösser and Schaffernicht (2012) highlight that there have been a multitude of approaches proposed for representing and assessing different elements of mental models. As mentioned by Schaffernicht and Grösser (2014), a variety of measures for structural complexity have been advocated in the literature: the number of variables, the number of links, the total, mean, and standard deviation of link delay, the link-to-variable ratio (i.e., the proportion of links to variables within any given CLD), the map density (i.e., the number of observed links divided by the total number of links theoretically possible, given the set of variables), the number of loops, the loop length (number of links in a loop), and the number of delays in the loop (e.g., Clarkson & Hodgkinson, 2005; Eden, 2004, 1992; Markoczy, 1997). However, Schaffernicht and Grösser note that the dispersion in the literature of different methods for evaluating mental models has led to incompatible findings that impede learning in the SD community. They therefore developed a comparative analysis approach for assessing structural differences between mental models at element level (factors and causal links), loop level (feedback behaviour), and model level (dynamic behaviour) (Schaffernicht & Grösser, 2011). Specifically, the method aims to assess the degree of similarity between either models of different actors or between different versions of a model of one subject before and after an intervention. The method is based on three measures: (i) Elements Distance Ratio, the differences between two maps considering variables and causal links; (ii) Loop Distance Ratio, the similarity between each pair of loops for the two compared maps; (iii) the Model Distance Ratio, the average of all loop distance ratios (Schaffernicht & Grösser, 2011, 2014).

It is also possible to identify different features of a causal map by representing it with an adjacency matrix, based on the theory of directed graphs (Harary et al., 1965). This is often used in the analysis of directed graphs from the area of Fuzzy Cognitive Maps (Özesmi & Özesmi, 2004). The adjacency matrix is a squared asymmetric matrix *n x n*, where *n* is the number of elements in the corresponding causal map. The content of each cell lying at the intersection of a row and column specifies the existence or non-existence of a causal relationship, namely when a connection exists between two variables (from rows to columns), the value of the link weight is coded in the range [−1, 1], otherwise the matrix cell has value 0. According to Langfield-Smith and Wirth (1992), when comparing the causal maps of two individuals or group of stakeholders using adjacency matrices, three types of difference can be identified: (i) Existence or non-existence of elements, (ii) Existence and non-existence of beliefs, (iii) Identical beliefs held with differing strengths. Furthermore, Eden (2004) presents seven ways for exploring CMs, each of which give an insight into ways of analysing the issue or problem, namely “islands” of themes (clusters without accounting for hierarchy), networks of problems (clusters accounting for hierarchy), “potent” options (external influences to multiple areas of the CM), virtuous and vicious circles, central concepts, simplifying the issue to the most connected problem, and shape (in terms of breadth vs. depth). Lastly, Valcourt et al. (2020) mention that is not possible to directly compare the dynamic behaviour of two models, despite the fact that they both draw on a relatively common set of factors and causal links.

Overall, the lack of unanimous alignment on more than one factor or causal link highlights the degree to which stakeholders have substantially different conceptualisations of how key elements of their local systems are connected, even if there is broad agreement on the elements themselves. Thus, the mere mention of a given factor by multiple interviewees does not indicate an alignment of interviewees’ understanding of the causal links between factors.

Lastly, the tedious nature of the merely quantitative comparison approaches raises questions about their replicability in different contexts (Schaffernicht & Grösser, 2014), especially when teams are not trained teams and the approaches are stakeholder-focussed approaches. In this sense, the drawbacks of applying purely quantitative approaches for comparing causal maps mainly concern the anatomy of participative approaches and type of outputs needed. For instance, in the case of extended participative SD modelling process aimed at supporting a collaborative decision-making process, there is often the need of effectively transferring knowledge back to the stakeholders. The quantitative analysis does not easily translate findings into the thinking of the stakeholders. Furthermore, the thematic aspect representing how stakeholders understand a problem/system is generally not present, reducing the effectiveness of the participatory application. Indeed, quantitative comparisons focus on the structure and existence of a loop, variable, or connection, and not on what stakeholders pay attention to, in terms of themes and topics. Within our stakeholder-focused approach, the need to translate the comparative analysis into independently usable knowledge was pivotal; the objective was to show areas of interest or themes to easily discuss, with different groups of stakeholders, what the groups’ similarities and differences of problem framings and boundary perceptions are, in order to reach a shared understanding of the problem under investigation. Furthermore, from an operational point of view, a quantitative comparison was considered as not always feasible in case of multiple large and highly connected CLDs with >100 variables each. At the end, as underlined by Langfield-Smith and Wirth (1992), quantitative measures could be used to enhance, not replace, qualitative analysis.

### 2.3 Reflections on differences in system boundary perceptions

A first basis for understanding different mental models in stakeholders’ perception of system boundaries is provided by the theory Boundary Critique. It looks at boundaries, values, and marginalization processes, which usefully enhance the reflections on understanding differences and comparing CLDs in multi-stakeholder decision-making. As underlined by Midgley and Pinzón (2011), the literature on Boundary Critique deepens considerations on system boundaries, including: (i) the link between people’s value judgements (about what purposes it is right to pursue) and boundary judgements (what they see as relevant to those purposes); (ii) how situations involving people who make different value and boundary judgements can result in entrenched conflict, (Midgley, 1992); and (iii) how people can reframe their understandings of a problem, thereby making progress in addressing it, by exploring different perspectives on their boundaries of concern (Midgley, 2000; Ulrich, 1983).

Churchman (1970) draws attention to the importance of setting boundaries during an intervention, considering that boundaries are the result of value judgements that indicate how problems are managed, what information is considered relevant and what is considered superfluous (Foote et al., 2007). Midgley and Pinzón (2011) claimed that prior to the work of Churchman, most writers on systems thinking (e.g., von Bertalanffy, 1968) assumed that the boundaries of a system are ‘given’ by the structure of reality. The convention in SD is to set boundaries through considerations of endogeneity: if an element is significant to the causality, it should be included (Midgley, 2000). The theory of Boundary Critique considered the boundaries always linked to value judgements (Ulrich, 1987), and this reflection well articulate the need of systematically comparing different stakeholders’ causal maps around the same issue. For Churchman (1970), boundaries define the limits of the knowledge that is to be taken as pertinent in an analysis and the people who generate that knowledge who also have a stake to improve the system (Midgley et al., 1998). While we can always improve on our current understandings of a problem by widening the boundaries of analysis and wide-spreading stakeholder involvement, sweeping in a variety of relevant perspectives (Ulrich, 1987).

Furthermore, the literature on Boundary Critique mentions the marginalization process (Midgley, 2000). Namely, given two groups, if one group makes a narrow boundary judgement and another makes a wider one; therefore, broadening boundary perceptions can be useful in supporting problem understanding and decision-making.

### 2.4 The contribution of the attention-based view

The notion of mental models shares similarities with the concept of organisational cognition, even though they originate from different literatures. Within organisational cognition, the notion of attention is well suited to support the understanding of the selectiveness of diverse groups’ mental models, problem frames and system boundaries. The Attention-Based View regards attention as the central organisational process on which decision-making is based (Simon, 1947; Ocasio, 1997, 2011).

Decision-makers in organisational contexts are believed to have limited attentional capability (Ocasio, 1997), affected by a multitude of factors, including organisational goals, strategy and identity, as well as individual or collective schemas and mental models (Suzuki, 2017). The stimuli from the environment of decision-making are actively reshaped by their attendants, in order to be accessible. These stimuli are interpreted through mental models, in order to draw inferences that explain the causes and effects of those stimuli and permit mental simulations of the outcomes of alternative plans of action or scenarios (Hoffman & Ocasio, 2001).

Specifically, the Attention-Based View of organisations (Ocasio, 1997) sheds light into what determines attentional processes and how this affects decision-making and action. It suggests how structure, process and context determine which environmental stimuli decision-makers attend to. These components could therefore contribute to reshaping mental models of problem frames and system boundaries, and respective decision-making. Thus, within the context of this paper, participatory causal mapping is expected to enable the elicitation of mental models with the underpinning cognitive, structural, and contextual factors. In addition, the active shaping of the cognitive, structural, and contextual factors during the participatory process may help adopt a systems perspective, creating a shared understanding of the system under consideration.

Drawing on these reflections, this paper aims to support a collaborative decision-making process, developing a methodology to compare large CLDs in a participatory multi-stakeholder setting, taking into consideration differences and similarities of groups’ system boundaries collected during a divergent phase before creating a shared understanding of the system under consideration. We uses this methodology to portray the thematic content of the CLDs to aid mutual understanding.

This paper methodologically innovates by combining well-established procedures from qualitative research and particularly open coding used in grounded theory (Glaser & Strauss, 1967; Strauss & Corbin, 1998) with methods to analyse and compare CLDs and more generally causal maps. With this multi-methodology lens, conceptualisation of SD models is often supported by a grounded theory approach to get from textual data to model structure (e.g. Kim & Andersen, 2012; Yearworth & White, 2013). In contrast to this, this paper uses thematic analysis not for the conceptualisation of the model but for an analysis of its content (see Sections 3 and 4 for details).

## 3 Methodology

### 3.1 The case study context: Thamesmead

Thamesmead is an area spanning for about 750 ha along over three miles of the Thames river, in South-East London (UK). Administratively covering two of the 32 boroughs constituting London, about 45,000 people distributed in 16,000 households reside in the area, with a significantly lower population density than inner London, and twice the amount of green space per person than the London average. Thamesmead is characterised by a rich network of parks spreading over 150 ha of green space and 32 ha of water, consisting of five lakes, seven km of canals, 5 km of Thames waterfront, plus 14 sites of nature conservation interest (Peabody, 2019).

The built environment mainly consists of concrete social-housing stocks, built from an inhospitable marshland since the mid-1960s, when the Greater London Council acquired the land with promises to build one of Britain’s most ambitious post-war housing projects. The masterplan was never fully realised, but perhaps its greatest legacy was the sheer amount of open space and waterways that, even today, Thamesmead residents say is one of the top reasons they like the area (Ford & Baikie, 2018).

Nowadays, Thamesmead is affected by a number of vulnerabilities. With only a third of adult residents in full-time employment (ONS Census 2011), Thamesmead represents one of the most deprived neighbourhoods in England (HMRC 2019), with significant child poverty (HMRC 2016) and about 40% of the population living in privately or socially rented accommodations (Peabody, 2019).

Since 2014, 65% of the Thamesmead housing estate has been owned by the Peabody Trust, and a masterplan will soon outline a 30-year regeneration vision, with a £1 billion investment to capitalise on the opportunities of the area. The Peabody Housing Association now oversees the management and maintenance of the area.

### 3.2 The qualitative modelling processes

Within the Thamesmead case study, the objectives of the qualitative modelling process were: (i) to bring together Thamesmead institutional stakeholders to jointly scope the focus of this work in the area of blue and green space, sustainability and health, (ii) to build different Thamesmead CLDs around the identified shared concern to capture the system boundaries for each group of stakeholders, included residents, (iii) to jointly understand differences in the stakeholders’ perceptions of system boundaries and their potential effects on decision-making, and (iv) to collectively agree on the focus of the following modelling steps.

**Fig. 1:**
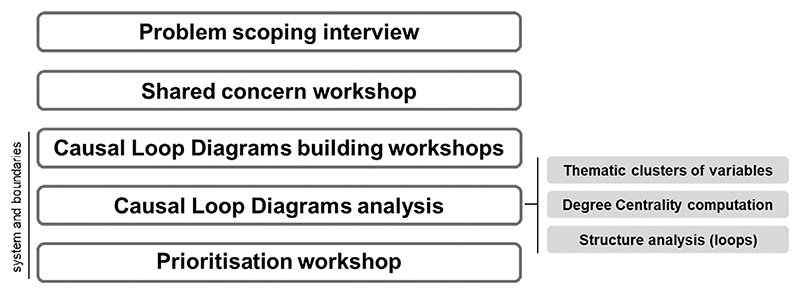
Participatory System Dynamics Modelling Process.

This section presents the steps of our participatory qualitative SD modelling process (Fig. 1) for eliciting and analysing the differences in problem frames and systems boundaries, whereas the case study results are described in Section 4 and discussed in Section 5.

### 3.3 Eliciting the differences in systems boundaries and CLDs building

A multi-step methodology was used to build the CLDs, through the following activities summarised in Table 1.

1. **Problem scoping interviews**. A series of short, semi-structured interviews supported institutional stakeholder engagement and data collection for background information. The interviews aimed to gather information on perceptions of the problems affecting the case study, perceptions of the evolution over time of the problems, perceptions of the causes and consequences, current policies and strategies. The main outcome of this step is an interlinked list of problems representing the starting point for the workshop discussion and a preliminary stakeholder analysis for identifying interviewees’ role and goals. For the selection of the stakeholders to be involved, “snowballing” or “referral sampling” (Reed et al., 2009) was implemented. During the interviews, each interviewee suggested the involvement of other stakeholders considering their role and expertise.
2. **Shared concern workshop**. This problem identification workshop brought together key institutional stakeholders and collaborators to jointly scope the focus of this work within the case study. Using the background information collected via interviews, this workshop discussed the most pressing problems to understand the direction of the future modelling activities. The main outcome of this workshop was consensus over a shared concern (namely a shared representation and formulation of a “problem” which serves as a representation or “recall” of the different concerns and stakes carried by the different stakeholders, see Irene Pluchinotta et al., 2019; Ostanello & Tsoukiàs, 1993).
3. **System and boundaries - CLDs building workshops**. A set of workshops aimed to build several CLDs around the selected shared concern was carried out; each CLD represented the perception of the system boundaries for a group of stakeholders. A Group Model Building technique was used. Similarly to Problem Structuring Methods, Group Model Building rests on a combination of modelling and facilitation, seeking to involve clients in the modelling process (Franco & Rouwette, 2011; Scott, Cavana & Cameron, 2016). CLDs building workshops were carried out in face-to-face or online settings (see Zimmermann et al., 2020 for insights on organizing online workhops). The workshops started with an elicitation of core variables related to the shared concern, using the Variable Elicitation script (Scriptapedia Wikibooks contributors, undated). Then, about 4–5 hours of causal mapping, similar to the Eliciting Initiating and Elaborating a "Causal Loop Diagram" or "Stock and Flow" model followed (Scriptapedia Wikibooks contributors, undated). In the context of the COVID-19 pandemic and the related travel restrictions and social distancing mandatory measures, we divided the workshop with one group into three two-hour online sessions in which a shared screen in Vensim® software replaced the large physical whiteboard for model building. These core activities were preceded by an introductory presentation of the objectives of the workshop, and concluded with the plan of the next stages.
4. **System and boundaries - Causal loop diagram analysis**. A novel analysis of the content of the CLDs followed, which is described in Section 3.4.
5. **System and boundaries - Prioritisation workshop**. The third workshop aimed to recognise and discuss differences and similarities in the perceptions of system boundaries. The results of the CLDs semantically meaningful coding, analysis and comparison were presented, and a group discussion supported the development of a joint understanding between different stakeholder groups. Participants discussed the main unexpected and known interrelationships. This involved a presentation of different groups’ focus in colour and themes, voting on favoured areas of analysis, as well as a substantial discussion to reach a consensus over the modelling priorities, collectively defining the focus of the quantitative SD model.

See the online Supplementary Materials for the agenda of each workshop.

### 3.4 Analysing differences in system boundaries and the CLDs comparison

Methodologically, this paper innovates by combining well-established procedures from qualitative research, and particularly the open coding used in grounded theory research (Glaser & Strauss, 1967; Strauss & Corbin, 1998) or inductive thematic analysis (Braun & Clarke, 2006), with methods to analyse and compare CLDs and more generally causal maps graphs. This also allows a novel focus on the thematic content of CLDs in addition to their structure and individual components. Coding is a research method used to identify and label features in a data set, with the aim of tracing similarities and differences; it “opens up to the text and expose the thoughts, ideas, and meanings contained therein” (Strauss & Corbin, 1998 p.102). The identified codes with overlapping meanings are eventually clustered into homogeneous themes (Braun & Clarke, 2006).

The structured CLDs comparison was an innovative combination of rigorous procedures from qualitative research with methods to analyse CLDs and formally compare graphs. Firstly, we investigated the CLDs’ key features and stakeholders’ attention, coding each variable in the CLDs to identify clusters of semantically meaningful themes (henceforth called also thematic clusters or clusters) using an inter-coder comparison activity; operatively three researchers associated a theme to all the variables, first individually and then collaboratively until consensus was reached. Secondly, we analysed the structure of the CLDs through an examination of the main interrelationships and loops, and the computation of the Degree Centrality for each map (i.e., a mathematical indicator from Graph Theory identifying central items in a causal map).

The analysis of the CLDs produced by the different stakeholder groups consists of several phases aimed to evaluate and compare the CLDs structures and key features:

1. **Initial coding and identification of thematic clusters of variables**. As CLDs are maps made of variables and arrows, we interpreted variables as concepts that can be aggregated into thematic clusters. The definition of thematic clusters has been carried out through a consensual inter-coder comparison activity with three independent coders. Firstly, we decomposed the CLD into a list of variables, then we used open coding of model variables, and each coder independently identified and named the clusters. This sectoral analysis was done at a high level of granularity, using up to three levels of sub-categories. In a first inter-coder comparison, many similarities could already be observed between the coders, but the granularity of identified cluster in different areas, e.g., water aspects vs. social aspects, also showed the coders’ disciplinary differences. The three coders discussed non-converging results in a collaborative way during several rounds of a consensus-reaching activity until full consensus on the naming of thematic cluster was achieved. Often, the finest level of granularity was chosen.
2. **CLD re-coding**. After the first inter-coder comparison of thematic cluster names, the coders re-coded the CLDs to investigate the CLDs’ key features, i.e., what themes, concepts and parts of the system our stakeholder groups paid attention to. We iteratively discussed remaining discrepancies until we reached full consensus on the attribution of all variables to a thematic sector for all CLDs. This process somewhat overlapped with the agreement on the naming of thematic cluster.
3. **Degree Centrality computation**. Considering the CLD as a directed graph, and following the principles of graph theory, we computed the Degree Centrality for each variable to identify the key variables of the CLDs, showing a prominent existing way to further identify similarities or differences between the maps.
4. **Thematic comparison**. The main objective of the CLDs comparison is to identify the differences in understanding the system boundaries of the shared concern within our case study, in order to support further discussions. To complement existing mathematical ways to compare CLDs, we colour-coded the CLDs based on the identified thematic cluster. We used same-colour shading of variables belonging to the same thematic sector. This produced colourful visuals that intuitively portray the thematic content of the CLDs, and thus the diverse groups’ mental models. Such a comparison offers the differences and similarities of the thematic cluster, underlining factors influencing the shared concern and representing relatively separable parts of the shared concern.
5. **CLD structure analysis**. Considering the Degree Centrality and more generally each CLD’s structure, the main interrelationships and key loops have been described and then discussed with the stakeholders. A high Degree Centrality indicates that a variable is highly linked to other variables. Therefore, feedback loops including variables with higher Degree Centrality could potentially imply higher chances for triggering/reverberating changes in the wider system when a change occurs within the loop. Therefore, in our large CLDs, loops including variables with higher Degree Centrality helped the selection of loops we discussed with stakeholders and were prioritised for the analysis.

In relation to the inter-coder comparison activity, we propose that the identification and validation of the CLD cluster can be managed as a qualitative coding activity. Specifically, when using qualitative coding techniques, establishing inter-coder reliability is a recognised method of ensuring the trustworthiness of the study when multiple researchers are involved with coding (MacPhail et al., 2016). The inter-coder reliability assessment can yield numerous benefits for qualitative studies, which include improving the systematicity, communicability, legitimacy and transparency of the coding process; promoting reflexivity and dialogue within research teams; and helping convince diverse audiences of the trustworthiness of the analysis. Moreover, two additional independent coders are frequently recommended as necessary to establish inter-coder reliability. In order to have a more generic approach allowing also the comparison of CLDs, the thematic clusters do not account for the hierarchy of the map, and they are used instead to explore the content of each cluster as already suggested by the ‘islands of themes’ of Eden (2004). However, the main differences are: (i) the methodology used for identifying such thematic clusters, grounded in coding processes often used in qualitative research and taking into consideration the consistency of the study, and (ii) their direct use in the comparison and discussion with stakeholders, as described above.

The Degree Centrality calculation allows to identify the most important vertices within a graph, accounting for the complexity of its network of links (Axelrod, 1976). The analysis can take place by representing a map as an adjacency matrix (Harary et al., 1965) (see Section 2.2). The content of each cell lying at the intersection of a row and column specifies the existence or non-existence of a causal relationship, namely when a connection exists between two variables (from rows to columns), the value −1 or 1 is coded, otherwise the matrix cell has value 0. The elements in the matrix are read lexicographically “from row element to column element” to indicate the direction of the relationship. The Degree Centrality is defined as the summation of its in-arrows and out-arrows (e.g., Özesmi & Özesmi, 2004). The Degree Centrality of a node is an easy centrality measure to compute, counting how many neighbours a node has. As mentioned by Eden et al. (1992), it represents a powerful tool for a simple analysis of complexity. The higher the degree, the more central the node is. Out-degree and in-degree describe the aggregated strengths of connections respectively as row and column sums of absolute values (Papageorgiou & Kontogianni, 2012). This can be an effective measure, since many nodes with high degrees also have high centrality by other measures. Considering that the aim of our methodology is only to provide insights on the central variables as part of an extended multi-methodology including thematic comparison analysis for large CLDs, regardless of the connection length, the identification of the Degree Centrality was considered useful. Therefore, the Degree Centrality was used instead of other centrality measures, e.g.: (i) Closeness centrality of a node (the average length of the shortest path between the node and all other nodes in the graph); (ii) Betweenness centrality of a vertex (the number of times a node acts as a bridge along the shortest path between two other nodes); (iii) Eigenvector centrality (measure the influence of a node in a network, a node is). Our CLDs comparison methodology is based also on thematic analysis and it does not take into consideration the differences between longer and shorter paths in a graph; therefore, Closeness and Betweenness centrality were not considered relevant measures to include at this stage of the methodology development. Within our methodology, we looked at the complexity of the map considering only the immediate links of each node and we decided to identify the central variables independently by the length of their links (Golbeck, 2013). Moreover, in relation to the Eigenvector centrality, it differs from in-degree centrality because a node receiving many links does not necessarily have a high eigenvector centrality (it might be that all linkers have low or null eigenvector centrality). On the other hand, a node with high eigenvector centrality is not necessarily highly linked (the node might have few but important linkers) (Golbeck, 2013); however, the Eigenvector centrality compute is outside the scope of our methodology. Lastly, Eden and Ackermann (1998) and Eden (2004) introduce a so-called Domain Analysis, a measure of immediate links to a concept for seeking out the ‘nub of the issue’ and detecting the structural characteristics of issues. It calculates the total number of in arrows and out arrows from each node; that is described as its immediate domain. Those nodes whose immediate domain are most complex are taken to be those most central, indicating the richness of meaning of each particular statement (Bryson et al., 2004). With this in mind, our approach for comparing CLDs aims to ensure the replicability of the methodology regardless the use of specific software and the level of expertise of the analysts, thus using generally available measures. To conclude, we consider the combination of the thematic cluster analyses and the Degree Centrality computation a useful combination of qualitative and quantitative methodologies, namely, including a semantic characteristic into quantitative methods to compare causal maps emerging from groups of stakeholders. Furthermore, we focus on themes and present the Degree Centrality as a different and already existing analysis methods. On the other side, the thematic analysis based on quantitative research methods is innovatively applied for a CLD analysis. Indeed, we propose the thematic analysis and the Degree Centrality computation as different and complementary ways to understand focal areas of the CLDs.

The novel methodology for building and comparing CLDs within a collaborative and multi-stakeholder context has been applied to a case study presented in the next section, focusing of the urban regeneration of an area characterised by several vulnerabilities.

## 4 Results: the Thamesmead case study

### 4.1 Workshops outcomes

#### Problem scoping interviews and shared concern workshop

The first two activities aimed at identifying the shared concern to focus on during the subsequent interaction stages, and they also elicited problems as seen from the perspectives of the participating stakeholders, first individually and then collaboratively. The resulting set of problems were grouped by the researchers into the following four main clusters (see the online Supplementary Materials for the full list), i.e.: (i) water, including physical (e.g. poor water quality, flood risk) and social problems (e.g. low use of blue space); (ii) pollution, largely overlapping with problems regarding water, and including traffic or risks for population health; (iii) climate change, with the acknowledgement of the effects from changes in weather conditions; (iv) connectivity and accessibility, mostly related to infrastructure, use of space (e.g. green and public ones in general) and perceptions (e.g. of quality and safety). A fifth cluster on governance and maintenance, related to the complexity characterising the local area (e.g., demographics, stakeholders, maintenance), was absent in the interview analysis and emerged from the workshop.

The discussion about problems and connections in the resulting map facilitated during the workshop enabled the stakeholders, with the facilitation of the researchers, to collaboratively identify the main problem that the forthcoming SD modelling stage could contribute to address, namely “how to sustain and increase the quality of the built, blue and green environments to ensure long term stewardship”. The quality of the three environments plays a central role in the map, by bridging the five clusters of problems. Specifically, during the workshop, the need for a longer-term stewardship plan, which includes community engagement, maintenance, funding, and environmental strategies emerged, as well as the importance of a better understanding of the complex relationships between the stakeholders involved, also in view of shared responsibilities.

#### System and boundaries - CLDs building workshop: face-to-face and online settings

Following the identification of the shared concern, we intended to define the system and its boundaries through participatory CLD-building with the respective groups of stakeholders: (i) Researchers/Modellers (who were engaged in sustainable and resilient urban development in the case study and informed on the physical sub-systems in the wider Thamesmead model), (ii) Environment and Government (E&G), (iii) the social Housing Association (HA) and (iv) Thamesmead residents.

The modelling workshops with the first three groups were held either in person (first two groups) or on-line (third group, in response to COVID-19 restrictions). These followed the scripts described in Section 3.1. The CLD reflecting the view of the residents was generated from both interviews and literature. The individual semi-structured interviews engaged a purposive sampling of seven local representatives (identified by the researchers) and residents (providing availability to be interviewed after submitting a short online questionnaire). Relevant articles from the scientific and grey literatures were selected based on their focus on social dynamics in Thamesmead and related areas.

The CLDs resulting from the engagement of the Thamesmead stakeholders were subsequently analysed and compared, to identify differences in the perception of the system boundaries and problem formulation (see Section 3.2 for the methodology and Section 4.3 for the application). For the purpose and methodological aim of this paper, we present the comparison analysis of only two CLDs, i.e., stakeholder groups “Environment and Governance” (E&G) and “Housing Association” (HA).

#### System and boundaries - Prioritisation workshop

The last workshop intended to share the results of the comparison analysis of the CLDs generated from the previous set of activities, to inform the discussion on the theme to be prioritised in the subsequent stage of the project. Participants were shown the thematic analysis, some of the loops emerging across the CLDs selected on the basis of their relevance (e.g., variables with high Degree Centrality, loops crossing different cluster) and they were encouraged to comment on the presented insights, especially on surprising and unexpected interrelationships. The discussion covered: (i) the importance of modelling climate change aspects within the case study in order to investigate the impacts on the water system, e.g. flood resilience of the Thamesmead local community and buildings, water quality of the unique network of lake and canals for habitat and people; (ii) overlapping goals across the represented organisations amongst the participants, which suggested the benefits of a collaborative approach to the planning activities; (iii) the recreational use of space and the importance of identifying strategies for increasing residents’ interest in them, especially in the light of the diversity characterising the social groups in the investigated areas and the relevance attributed to blue and green space during the COVID-19 pandemic, during which the workshop was held.

In conclusion, although the discussion started with climate change, water quality and governance, participants agreed that the usability and use of the space were key elements to be investigated in the subsequent modelling activity. A recurrent concept was the need for a deeper understanding of dynamics leading to hindering local people in attending to natural space interconnected with concepts of governance as well as ownership. This concluded the first engagement phase with stakeholders and provided a rich basis for quantitative modelling.

### 4.2 Analysing the differences in system boundaries and the CLDs comparison

**Fig. 2:**
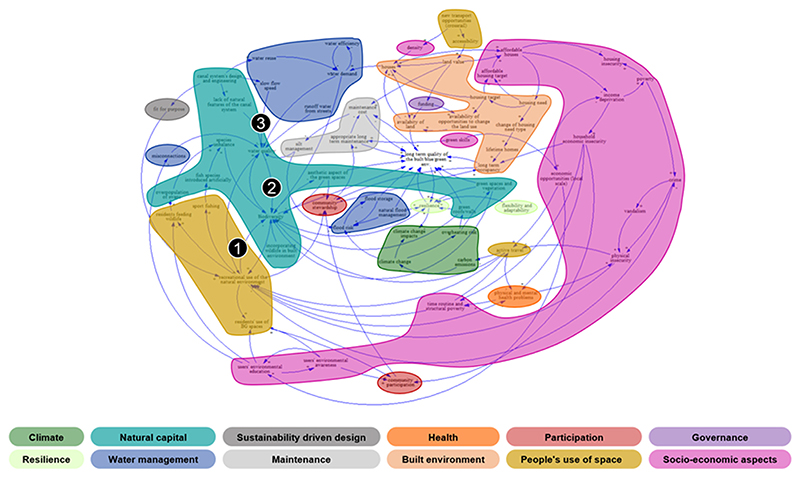
CLD built by the stakeholder group "Environment and Governance" (E&G) around the shared concern of the Thamesmead case study. The 1st order thematic clusters follow the colour code of Table 2 recalled in the bottom part of the figure. This paper discusses only on the 1st order thematic clusters. The variables with the highest Degree Centrality have been highlighted: 1) Recreational use of the natural environment, 2) Biodiversity, 3) Water quality. Coloured figures are available in the online version of the paper or upon request to the authors.

**Fig. 3:**
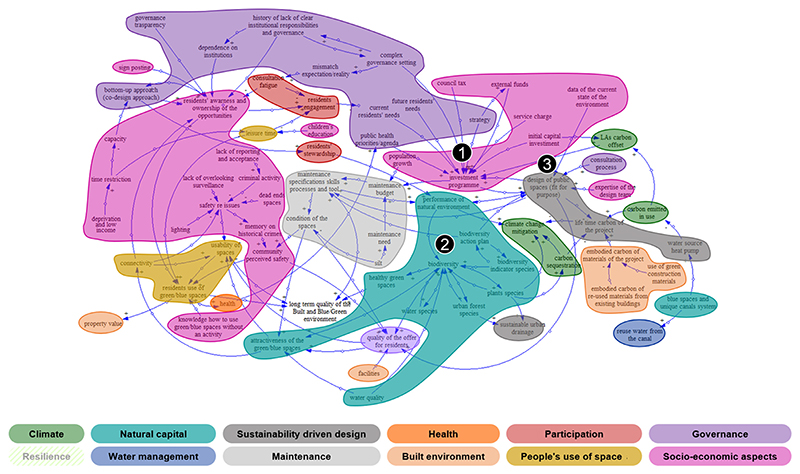
CLD built by the stakeholder group "Housing Association" (HA) around the shared concern of the Thamesmead case study. The 1st order thematic clusters follow the colour code of Table 2 recalled in the bottom part of the figure. This paper discusses only on the 1st order thematic clusters. The variables with the highest Degree Centrality have been highlighted: 1) Investment programme, 2) Biodiversity, 3) Fit for purpose driven design of public space. Note that resilience in with higher transparency because missing in the CLD. Coloured figures are available in the online version of the paper or upon request to the authors.

**Fig. 4:**
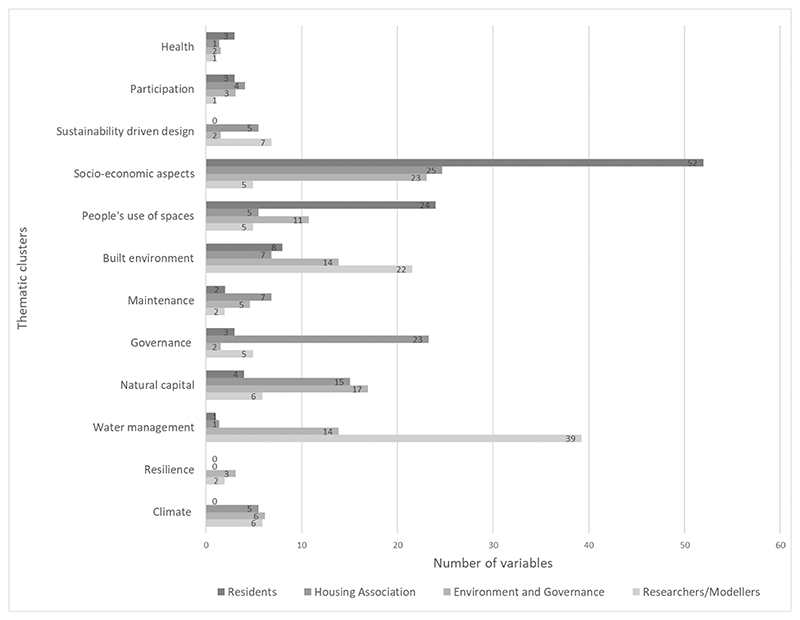
Number of variables for each cluster normalised on the total number of variables of each CLD. All the groups of stakeholders are represented.

Following the multi-step methodology for the thematic comparison described in Section 3.2, two final orders of thematic clusters were identified (Table 2) and the variables of each CLD were coded (Figs. 2 and 3). The CLD key features were then investigated, together with the analysis of the map structure using Degree Centrality (Table 3) and loops analysis. Fig. 4 shows a comparison of the number of variables belonging to the 1st order clusters normalised on the total number of variables of each CLD (including the groups of secondary focus in this paper). The normalised number of variables in each cluster is then used to support the discussion below.

Different granularities of coding are reported in case subcategories. However, for the sake of brevity, only the first order of themes is described for both CLDs under consideration (see the online Supplementary Materials for the non-coded CLDs). The colour distribution, representing the semantically meaningful coding, already offers a preliminary visual insight of the cluster predominating in each CLD for the interpretation of the shared concern (namely “how to sustain and increase the quality of built, blue and green environments to ensure long term stewardship”) from both stakeholder groups, “Environment and Governance” (E&G) and “Housing Association” (HA). The thematic clusters colourful visualization aimed to intuitively portray the thematic content of the CLDs, highlighting the size of each cluster to support further discussion.

Considering the methodological purpose of this work and for the sake of simplicity, this case study discusses only on the 1st order thematic clusters. However, the distinction between different levels of granularity (1st and 2nd order) allowed to code closely to the detailed meaning of a variable at the 2nd order while also being able to establish an aggregated understanding at the 1st level.

In both CLDs, the ‘Socio-economic’ cluster covers a significant part, including concepts such as economic factors, residents’ knowledge and awareness, people’s vulnerability, safety and demographic aspects. Surprisingly, only E&G focuses on the housing aspect, introducing key concepts such as housing affordability. Within HA’s CLD, the variables related to the ‘Socio-economic’ sector describe the safety issues of the natural space, providing details on possible drivers related to the layout or to crime and perceived safety (e.g., lack of reporting and acceptance, memory of historical crimes). Economic factors, such as poverty and deprivation, are part of both CLDs. However, variables describing the ‘social aspects’ and ‘business and services’ themes are missing.

The ‘Natural capital’ theme is covered by a large sector in both CLDs; for instance, biodiversity is equally considered, but with different contents and interrelationships (species unbalance is described by E&G, while HA introduces biodiversity action plans and indicators). The HA group has an extended ‘Governance’ sector reflecting mostly the funding and investment programme, the governance complexity and the residents-focused agenda, while this is only represented by the variable ‘funding’ in the E&G CLD. On the other side, E&G includes a wide ‘Built environment’ sector mainly related to housing, but it describes the interrelationships between housing target, long-term occupancy and lifetime houses (variables influencing the related residents’ stewardship). These concepts are missing in the HA group where the ‘Built environment’ sector focusses on embodied carbon and facilities.

Both E&G and HA pay moderate attention to the ‘People’s use of public space’ sector. Specifically, E&G mentions that the blue and green space are used for recreation when there is an increase of residents’ environmental education or awareness. Furthermore, HA introduces the concepts of usability of space. In both cases, the lack of leisure time and time routine is mentioned as main barriers, while residents’ participation is seen as a possible driver.

The ‘Water management’ sector is more extended for E&G, considering water efficiency, misconnections, but also flood risk, while it is represented only by one variable in the HA’s system understanding (i.e., quality of the blue infrastructures that is present in both CLDs).

For HA, the ‘Sustainability driven design’ is more influential than in E&G (only one variable). In both cases, the ‘Maintenance’ aspects of the shared concern are described with similar variables, but E&G captures the long-term feature of the shared concern. In addition, the ‘Climate change’ thematic cluster is addressed in different ways: while HA focusses on mitigation and carbon sequestration, E&G emphasises climate change impacts on the natural environment. Lastly, ‘Resilience’ is considered only by E&G, while ‘Health’ is briefly mentioned in both CLDs (E&G also introduces the concept of mental health). In both CLDs, the ‘Participation’ cluster describing the local community engagement is represented by a few similar variables, but differently connected.

Afterwards, the Degree Centrality was computed for each variable of each CLD. The E&G’s CLD is made of 246 connections; the ‘Recreational use of the natural environment’ is the most central variable, from a structural point of view. ‘Biodiversity’, ‘Water quality’, and ‘Resilience’ are also characterised by a high Degree Centrality. The HA’s CLD consists of 220 connections, and ‘Investment programme’ is the most connected variable, followed by ‘Biodiversity’, ‘Fit for purpose driven design of public space’ and ‘Residents’ awareness and ownership of the local opportunities’ on the use of blue and green space. Surprisingly, the variable related to the jointly-selected focus of the next modelling activities, ‘Residents use of blue and green space’, has only a medium Degree Centrality for HA.

Within E&G’s CLD, ‘Socio-economic aspects’ and ‘Natural capital’ are the largest cluster (respectively 23 and 17 variables); ‘Built environment’ and ‘Water management’ are also described by a high number of variables (14), and the variables ‘biodiversity’ and ‘water quality’ (with a high Degree Centrality) are part of this sector too. It is worth noticing that the most linked variable ‘Recreational use of the natural environment’ is part of a less populated sector made of only 11 variables.

Similarly, for HA the ‘Socio-economic’ cluster is the largest one (25 variables) together with ‘Governance’ (23). While the governance aspects are barely explored by the other group, in HA it covers a significant part of the CLD. Indeed, the most interconnected variable (i.e., ‘investment programme’) is part of this sector, and it is connected via many in-arrows. ‘Natural capital’ is the third sector per size with 15 variables (‘biodiversity’ variable has a high Degree Centrality, and it is part of this sector). ‘Fit for purpose driven design of public space’ is the third most interconnected variable, but it is part of a sector with only 5 variables.

**Fig. 5:**
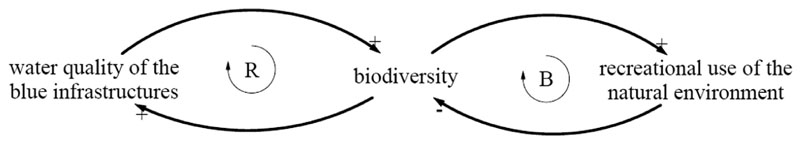
One of the loops from the E&G’s CLD, showing an unintended consequence seen by one group of stakeholders.

Considering the key loops, for the sake of brevity, it is worth mentioning the following one as an example of an unintended consequence that has been detected only by E&G (Figure 5). According to E&G, the more natural space are used, the less biodiversity is surviving, and the more biodiversity is present, the more the blue and green space are attractive, increasing their use for recreation (balancing loop, B). Furthermore, as biodiversity increases, so does water quality of the blue infrastructures, and vice versa (reinforcing loop, R). The combination of these loops is a “limit-to-growth” archetype, important for the development of effective strategies around the shared concern of the case study.

In conclusion, our analysis showed that all stakeholder groups have a rich understanding of the same shared concern, but that they also have clear differences in the detailed boundary perception of this shared concern, with potential effects on decision-making. The HA group’s attention to a multitude of factors in the governance sector allows them to potentially choose from a multitude of alternatives for deciding and acting on the shared concern, including co-design and consultation processes, transparency, a prioritisation of public health or future needs as well as their own strategy. Options seen by the E&G group contrastingly focus on the funding programme. Within the E&G group’s system boundaries, the residents’ participation is influenced by economic insecurity and environmental awareness/education, together with the actual use of blue and green space. On the other side, for HA, the latter aspects are not influencing participation, but consultation fatigue hampering engagement is introduced, along with the concept of stewardship affected by the use of co-design approaches. An integration between the two different system boundaries could allow the identification of a larger set of policy/strategies. In addition, the analysis also showed that both groups perceive somewhat similar system boundaries around the use of space, including biodiversity, economic or structural deprivation and perceived safety, potentially alleviating the joint decision on this focus area. The varied representation of clusters in each CLD possibly reflects the diversity of drivers of attention for each stakeholder group. Future research informed by the Attention-Based View could explore the organizational structures and procedures shaping attention patterns in order to clarify the diversity of clusters representation in the CLDs.

## 5 Discussion and conclusions

Scholars underlines that, in settings where multiple actors need to interact to tackle a complex issue, differences in perceptions of system boundaries may impact the effectiveness of collaborative decision-making. Each stakeholder sees a certain system from their own point of view and thus, with a narrow system boundary, fails to detect unintended consequences that others might be aware of, and only consider solutions within the perceived system. Within this context, this paper provides a methodological contribution for analysing a multiple perspectives about the system boundary to aid mutual understanding, by systematically comparing different stakeholders’ frames. It proposes an integrated approach for comparing CLDs, based on concepts from SD, OR (specifically Problem Structuring Methods and Boundary Critique), and qualitative research (i.e., semantically meaningful coding). Furthermore, it is inspired by the notions of organisational attention and Organisational Studies.

Methodologically, this paper combines SD and qualitative research in a different way than has been done before. Qualitative coding has so far been used to better conceptualise SD models (e.g., Eker & Zimmermann, 2016; Kim & Andersen, 2012). In contrast, we applied qualitative coding after a qualitative model has been built, to analyse and deeply understand the model content. This offers an additional way to understand model content, at a fine level of detail and complementary to an analysis of feedback relationships in the model. Moreover, this work expands on the methods for analysing CMs introduced by the OR literature, providing an approach for a semantic thematic analysis independently of the map hierarchy, and overcoming software dependency to ensure replicability. In addition, the thematic analysis and the Degree Centrality computation are used as complementary ways to understand focal areas of causal maps. The Degree Centrality computation is one of the established measures for CMs while a thematic analysis based on quantitative research methods is innovatively applied for a CLD analysis.

Furthermore, this paper adds a novel step to support collaborative decision-making that sits between stakeholder analysis and problem structuring, including another level of detail. It shows that a shared problem is still framed differently in a multi-stakeholder setting, bringing a higher risk for failure and conflict if differences remain misunderstood. This is particularly relevant for grand challenges and global problems (e.g., climate crisis, Sustainable Development Goals) because they require understanding of and collaboration between diverse stakeholder groups. Typically, in multi-stakeholder settings, only stakeholders’ goals and stakeholders’ power influence are analysed (e.g., Bendtsen, Clausen & Hansen, 2021). This higher level of detail allows SD and OR practitioners to formally compare and then visualise stakeholder groups’ system boundaries for a certain issue. The proposed integrated approach provides an intuitive way to visualise these differences, which is a starting point for shared understanding as well as more systemic perceptions of the system and, consequently, more systemic decision-making.

The paper also investigates how it is possible to support stakeholders through systems modelling. It provides the basis for creating shared understanding of the system and adopting a systems perspective and solutions that fulfil a multitude of sustainability criteria. Within the case studies, this allowed participants to see to which extent different stakeholders prioritised sustainability and health, plus social and environmental factors. For the sake of replicability, the paper fully describes a qualitative participatory SD modelling process aimed at defining the key aspects of a problem for each group of stakeholders (Sections 3 and 4). Firstly, a set of relevant issues was prompted, and the shared concern was jointly identified by the stakeholders. Secondly, the CLDs were built during the set of modelling workshops. Lastly, during a stakeholder workshop, differences and similarities in the perceptions of system boundaries were presented and discussed. Despite the challenges of the online setting, the last workshop represented a successful moment to discuss and overcome the differences and to collectively agree on the focus of the consequent quantitative SD modelling process.

We acknowledge that our work has multiple limitations and provides the basis for much further analysis. On the one hand, in order to be able to provide sufficient depth of reflection on the novel method of qualitatively analysing a CLD, we could only focus the description in this paper on two of the four stakeholder groups that we collected data from. On the other hand, the proposed approach allows practitioners to compare CLDs, not only between different groups, but it could equally allow to compare a single group/individual’s model at different points in time, mapping the evolution of incremental and transformational change. We thus recommend more research in this promising area.

Concerning the novel method itself, we want to emphasise that our suggested process breaks down complexity and allows for intuitive visualisation and communication. However, it does not focus on the system structure, such as comparing feedback loops, which are so important from a SD perspective. That is why we did not solely use the qualitative analysis but combined it with the Degree Centrality as well as with an assessment of core feedback loops. Further research could focus on: (i) the role played by the other centrality measures and the CLD geometry; (ii) the emerging literature on dominant loop analysis to create a link between our qualitative thematic coding method and quantitative analysing techniques.

In this paper we have explained that each stakeholder group’s CLD is a representation of the mental model of that group. Yet, the idea of a mental model at the group level is a somewhat contested concept (Kim, 2009). The CLDs represent what members of the group shared. We typically asked whether other group members agreed with the suggested structure, and our evaluation activity revealed that there was indeed broad agreement. While we considered this sufficient evidence to then speak of the group’s perception of the system and its boundary, future research could more deeply theorise the idea of a group’s perception in linkage with the causal mapping of system boundaries as done in our case study.

Future research could also further explore how our investigation of system boundaries relates to research on attention and framing, e.g., including the question of how perceived system boundaries relate to stakeholders’ familiarity with system elements, to their attributed importance and how this again links to the thematic clusters and their connectivity.

Lastly, this paper represents a first investigation in this direction and does not include considerations on what the differences between the framings in the CLDs stem from. A wider study on the role of values in relation to boundaries, central to the literature on Boundary Critique, could bring further insights and the causal perspective from the area of SD could help identify how understanding perceived causalities augments understandings of boundaries.

Overall, from a practitioners’ lens, this work proposes an intuitive and methodology for comparing CLDs based on the integration between a thematic analysis and quantitative methods, such as the centrality of the variables and the structure of the CLDs, which could easily be replicated by facilitators from different domains. The methodology has been beneficial in the context of our case study, characterised by time pressure to meet agreed deadlines, stakeholders’ limited availability to participate in meetings, large CLDs (approx. 100 variables for each CLD) representing highly interconnected issues, and most importantly, the need to communicate with the stakeholders the comparison results easily and plainly, in order to facilitate the participatory process and strengthen stakeholder engagement.

For the research community, this paper offers an additional effort to bridge three research domains, namely OR, SD, and Organizational Studies, around attention to and the framing of complex problems (Ocasio, 1997; Kaplan, 2008), often characterizing collaborative and multi-stakeholder decision-making. It highlights that the synergies on how the three communities deal with this specific issue is little investigated, and it proposes a novel approach. Furthermore, it brings an organisational perspective into group decision and multi-stakeholder settings.

## Supplementary Material

Supplementary Material

## Figures and Tables

**Table 1:** Steps of the participatory SD modelling process and workshops participants.

Steps of the participatory SD modelling process	No. of Participants	No. and type of Attendees	No. of facilitators and observers
Problem scoping interviews (1 h)	10	(1) Local Government (3) Environmental NGOs (1) Environment Agency (4) Housing Association (1) Social innovation NGOs	1 Facilitator 1 Observer from academia
Shared concern workshop (in person 3 h)	12	(1) Local Government (2) Environmental NGOs (1) Environment Agency (3) Housing Association (1) Social innovation NGOs	2 Facilitators 4 Observers from academia
System and boundaries – CLD building workshop Group “Environment and Governance” (E&G) (in person 6 h)	4	(1) Local Government (1) Water utility company (1) Environmental NGOs (1) Environment Agency	2 Facilitators 4 Observers from academia
System and boundaries – CLD building workshop Group “Housing Association” (HA) (3 online sessions of 2 h)	4	(4) Housing Association	2 Facilitators 3 Observers from academia
System and boundaries – Prioritisation workshop (online 3 h)	12	(2) Local government (1) Water utility company (2) Environmental NGOs (1) Environment Agency (3) Housing Association	3 Facilitators 3 Observers from academia

**Table 2:** List of the thematic clusters identified for the case study CLD and related colour code (coloured figures are available in the online version of the paper or upon request from the authors).

Thematic Clusters of Variables for the Thamesmead case study	
1st order	2nd order
Climate	Climate change Temperatme and rainfall Extreme events
Resilience	–
Water management	Water management -supply Water management -discharge Flooding
Natural capital	Blue spac.e Green space Biodiversity
Governance	Residents focused agenda Ftmding Responsibility
Maintenance	–
Built environment	Materials and constrnction Land use Housing
People’s use of space	Recreational use Active travel Accessibility
Socio-economic aspects	Housing affordability Economic factors Knowledge People’svub1erability Safety Population Social aspects Business and Services
Sustainability driven design	–
Participation	–
Health	–

**Table 3:** Degree Centrality of the CLDs variables.

Variables of the CLDs with the highest Degree Centrality	Groups	
	Environment and Governance (E&G)	Housing Association (HA)
1	Recreational use of the natural environment	Investment programme
2	Biodiversity	Biodiversity
3	Water quality	Fit for purpose driven design of public space
