# Supplementary material for: The importance of eliciting stakeholders’ system boundary perceptions for problem structuring and decision-making
Source: Eur J Oper Res. Author manuscript; Available in PMC 2024 Oct 21. (PMC7616725; doi:10.1016/j.ejor.2021.12.029)

**The importance of eliciting stakeholders’ system boundary perceptions for problem structuring and decision-making**

# Online Supplement

This section includes additional details related to the workshops developed during the qualitative modelling process described in Sections 3 and 4 of the manuscript.

Online Supplement Table O. 1. Problem scoping interviews for organizational stakeholders

| **#** | **Questions and prompts** | **Objective** |
| --- | --- | --- |
| **1** | (if appropriate) Could you, please, describe your role in your work related to Thamesmead? | Interviewee’s role and main objectives |
| **2** | According to your best knowledge, could you briefly describe the main environmental and water related problems affecting the area of Thamesmead? | Perceptions of the problems affecting the case study |
| **3** | What is the temporal dimension of these problems? For instance, was the mentioned problem persistent? When did the problem start and has it improved/worsened? | Perceptions of the evolution over time of the problems |
| **4** | What do you think is the most important problem the project should address? (Ranking order if necessary) | Research focus |
| **5** | (if appropriate) For each of the above-mentioned problem, who has this problem? And who is responsible for it? | Contribution to stakeholder analysis |
| **6** | For each of the above-mentioned problem, what are its causes? | Perceptions of the causes |
| **7** | For each of the above-mentioned problem, could you describe the main consequences? | Perceptions of the consequences |
| **8** | What are the main policies/strategies/framework that have been proposed, and are (or will be) implemented in order to deal with the above-mentioned problems? | Knowledge on policies and strategies for the area |
| **9** | (if appropriate) Is there anyone else you think we could usefully speak to? | Stakeholder snowballing |

Online Supplement Table O. 2. Agenda: Shared concern workshop

| **Time** | **Activity** |
| --- | --- |
| 30 min | Introduction and workshop objectives |
| 60 min | Problem scoping session |
| 10 min | Coffee break |
| 45 min | Core variables elicitation and behaviour over time |
| 15 min | Next steps and closing |
| 5 min | Evaluation |

Online Supplement Table 0. 3. Agenda: CLDs building workshops, face-to-face meeting, group “Environment and Governance”

| **Time** | **Activity** |
| --- | --- |
| 15 min | Introduction and workshop objectives |
| 60 min | Variable elicitation session |
| 45 min | Lunch |
| 120 min | Creating Causal Loop Diagram – Session 1 |
| 15 min | Coffee break |
| 60 min | Creating Causal Loop Diagram - Session 2 |
| 10 min | Closing and next step |
| 5 min | Evaluation |

Online Supplement Table O. 4. Agenda: CLDs building set of three workshops, online meetings, group “Housing Association” (2 hours each session) (see Zimmermann et al., 2020)

| **Time** | **Activity** |
| --- | --- |
| 10 min | Introduction (welcome and workshop objectives, recap of previous work/session) |
| 20 minutes | (Short) Variable elicitation session (in 1^st^ session) |
| 85 minutes | Creating Causal Loop Diagram (105 minutes in 2^nd^ and 3^rd^ session) |
| 5 min | Closing and next step |
| 5 min | Evaluation |

Online Supplement Table O. 5. Agenda: Prioritisation workshop (see Zimmermann, N. et al., 2020)

| **Time** | **Activity** |
| --- | --- |
| 5 min | Relevant sectors poll – Session 1 |
| 20 min | Introduction |
| 30 min | System boundaries - Presentation of the results |
| 25 min | System boundaries – Discussion Session 1 |
| 5 min | Coffee break |
| 30 min | System boundaries – Discussion Session 2 |
| 15 min | Relevant sectors poll – Session 2 |
| 40 min | Focus of the simulation - Discussion |
| 5 min | Closing and next step |
| 5 min | Evaluation |

Online Supplement Table O. 6. List of the most recurrent items emerged during the “problem scoping interviews” and presented at the beginning of the “shared concern workshop” (Water W, Pollution P, Climate Change CC, Connectivity and Accessibility C&A)

| **Problems** | **Clusters** |
| --- | --- |
| Thamesmead was built on a marsh land | W |
| Artificial nature of the network of canals and lakes | W |
| No natural flow in the canal network | W |
| High silt content | W, P |
| Low oxygen content | W, P |
| Misconnected households | W, P |
| Combined sewer system | W, P |
| High volume of traffic | P |
| Low natural connectivity | C&A, W, P |
| Degraded ecological habitat | C&A, W, P |
| Risk for population health | W, P |
| Low usage of blue areas | C&A, W, P |
| Low usage of green areas | C&A |
| Low usage of public space | C&A |
| Low social connectivity | C&A |
| Fragmented area | C&A |
| Lack of friendly pedestrian paths and spaces for recreational activities | C&A |
| Flood risk | CC |
| High flow | CC |
| Change of weather condition | CC |

Online Supplement Figure O. 1. CLD built during the workshop, group “Environment and Governance”


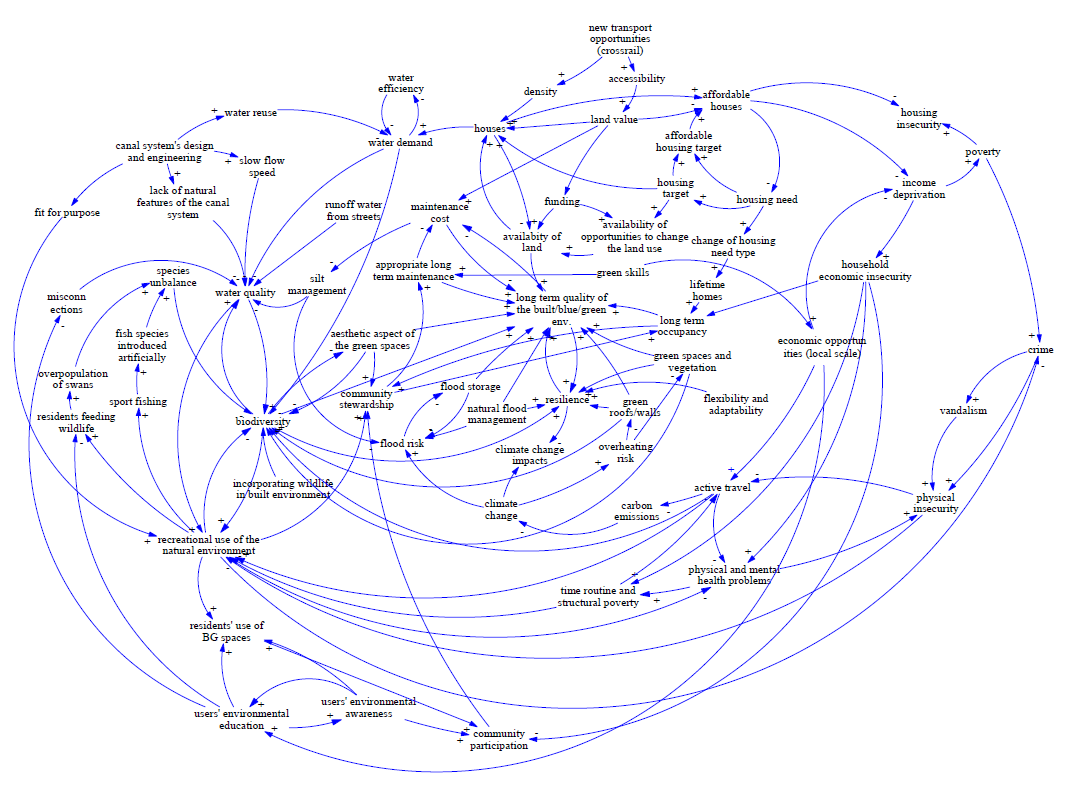


Online Supplement Figure O. 2. CLD built during the workshop, group “Housing Association”


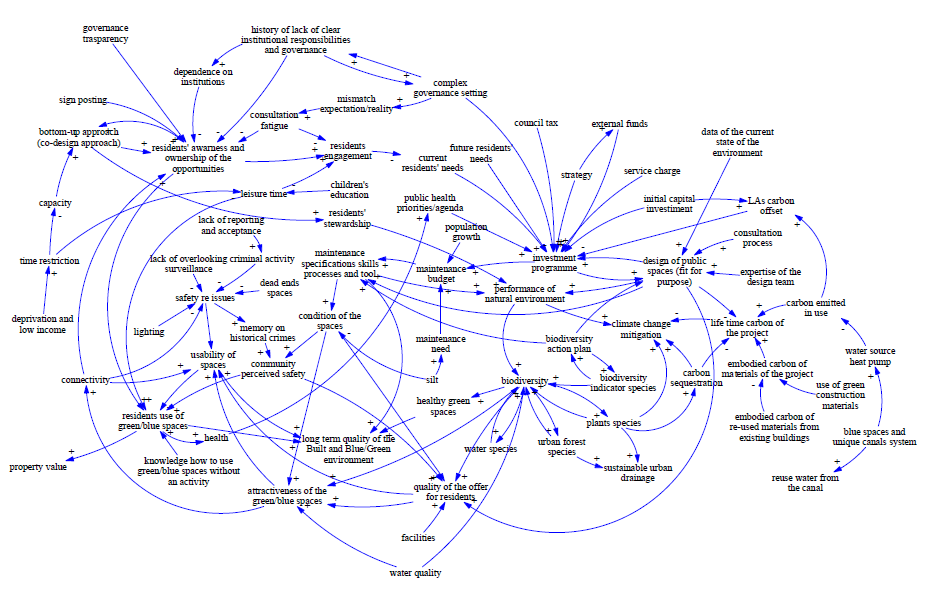

Supplement: Supplementary Material [file EMS199297-supplement-Supplementary_Material.docx]
